# Supplementary material for: MetaRibo-Seq measures translation in microbiomes
Source: Nat Commun. 2020 Jun 29;11:3268. doi: 10.1038/s41467-020-17081-z (PMC7324362; doi:10.1038/s41467-020-17081-z)
Supplement: Supplementary file 10 — Supplementary Data 7 [file 41467_2020_17081_MOESM10_ESM.zip › File2/Confidence_High_Taxonomy/409463_out.krona.html]

Javascript must be enabled to view this page.

members
magnitude
magnitudeUnassigned
count
unassigned
taxon
rank

409463\_out

41

2
41
superkingdom

phylum
37
1239

186801
36
class

order
36
186802

541000
35
family

1898205
species

SRS011134\_contig\_number\_27364SRS015663\_contig\_number\_14522SRS017701\_contig\_number\_4058SRS017916\_contig\_number\_13638SRS018541\_contig\_number\_8930SRS020394\_contig\_number\_5273SRS021948\_contig\_number\_35329SRS022609\_contig\_number\_44725SRS045004\_contig\_number\_21105SRS048164\_contig\_number\_28491SRS049446\_contig\_number\_6897SRS049959\_contig\_number\_45607SRS049995\_contig\_number\_31940SRS052697\_contig\_number\_39618SRS058070\_contig\_number\_15538SRS063518\_contig\_number\_26028SRS064757\_contig\_number\_16557SRS075773\_contig\_number\_25227SRS078176\_contig\_number\_19799SRS101376\_contig\_number\_16489SRS1041092\_contig\_number\_14940SRS1041133\_contig\_number\_20364SRS1041136\_contig\_number\_22294SRS1041141\_contig\_number\_12956SRS1041145\_contig\_number\_36268SRS104400\_contig\_number\_21895SRS143895\_contig\_number\_9433SRS144537\_contig\_number\_20481SRS147271\_contig\_number\_15069SRS147557\_contig\_number\_7959SRS148196\_contig\_number\_34088SRS149784\_contig\_number\_12340SRS150029\_contig\_number\_18215SRS971276\_contig\_number\_282
34

genus
1
216851

1971605
1

SRS078419\_contig\_number\_33207
species

216572
1
family

genus

SRS012849\_contig\_number\_19326
1
459786

1263000
species
1

SRS018836\_contig\_number\_3443

phylum
4
1224

1236
class
4

order
4
91347

543
4
family

570
genus
4

573
species
4

SRS015782\_contig\_number\_3810SRS019161\_contig\_number\_35956SRS051031\_contig\_number\_23205SRS147346\_contig\_number\_33576
